# lncRNA PVT1 and its splicing variant function as competing endogenous RNA to regulate clear cell renal cell carcinoma progression

**DOI:** 10.18632/oncotarget.19743

**Published:** 2017-07-31

**Authors:** Tao Yang, Hui Zhou, Peijun Liu, Libin Yan, Weimin Yao, Ke Chen, Jin Zeng, Heng Li, Junhui Hu, Hua Xu, Zhangqun Ye

**Affiliations:** ^1^ Department of Urology, Tongji Hospital, Tongji Medical College, Huazhong University of Science and Technology, Wuhan 430030, PR China; ^2^ Hubei Institute of Urology, Wuhan 430030, PR China; ^3^ Department of Urology, Jingzhou Central Hospital, The Second Clinical Medical College, Yangtze University, Jingzhou 434020, PR China

**Keywords:** PVT1, ccRCC, ceRNA, alternative splicing, miR-200s

## Abstract

Long non-coding RNAs (lncRNAs) exert critical regulatory roles in the development and progression of several cancers. Plasmacytoma variant translocation 1 (PVT1), an lncRNA, was shown to be upregulated in clear cell renal cell carcinoma (ccRCC) in our study, while Kaplan-Meier curve and Cox regression analysis showed that high expression of PVT1 was associated with poor overall survival (OS) and disease free survival (DFS) in ccRCC patients. *In vitro* experiments revealed that PVT1 promoted renal cancer cell proliferation, migration, and invasion, while *in vivo* studies confirmed its oncogenic roles in ccRCC. Further bioinformatic analysis and RNA immunoprecipitation revealed that PVT1 could function as an oncogenic transcript partly through sponging miR-200s to regulate BMI1, ZEB1 and ZEB2 expression. Besides, a novel splicing variant of PVT1 lacking exon 4 (PVT1ΔE4) was found to have a higher expression in ccRCC and could also promote cell proliferation and invasion as the full-length transcript did. Besides, SRSF1 decreased the inclusion of exon 4 of full-length transcript and increased the relative expression of PVT1ΔE4 in ccRCC. Mechanistic investigations indicated that PVT1ΔE4 could also upregulate the expression of BMI1, ZEB1 and ZEB2 through interacting with miR-200s. Our study helps reveal new molecular events in ccRCC and provides promising diagnostic and therapeutic targets for this disease.

## INTRODUCTION

Renal cell carcinoma (RCC) is one of the ten most common cancers, with approximately 202,000 newly diagnosed cases and 102,000 deaths worldwide annually [1]. RCC comprises a set of highly heterogenous malignancies in kidney, the most common subtype of RCC is clear cell renal cell carcinoma (ccRCC) which accounts for 75–80% of all renal cancers [2]. Apart from high rates of local invasion, metastasis and mortality, ccRCC is also refractory to traditional radiotherapy and chemotherapy, which makes its clinical management a thorny problem [2–4]. Although Fuhrman nuclear grade has been widely utilized to evaluate the severity and prognosis of this disease, reliable and effective biomarkers for early diagnosis and prognostic prediction are still lacking for ccRCC [5]. Better understanding of molecular events and underlying mechanisms involved in the carcinogenesis of ccRCC may provide us effective therapeutic targets and predictive biomarkers which are instantly required.

Long noncoding RNAs (lncRNAs) are a class of RNA transcripts which are longer than 200 nucleotides, evolutionarily conserved, and devoid of protein-coding potential [6]. Recently, emerging studies have shown that lncRNAs are frequently deregulated in various tumors and exert multiple functions in a wide range of biological processes, such as proliferation, apoptosis, cell cycle arrest, cell migration and invasion [7, 8]. By mining the lncRNA expression profile of ccRCC patients in The Cancer Genome Atlas (TCGA) database, we sorted out a panel of well-characterized functional lncRNAs including Plasmacytoma variant translocation 1 (PVT1). Containing 9 exons, PVT1 is a 1,957 bp linear lncRNA encoded by human PVT1 gene which is located at 8q24.21. It was found to be upregulated in certain human tumors [9], and some studies also revealed its correlations with pathologic stage, lymph node metastasis, and even survival rate in multiple cancers [10–12]. These findings advocated PVT1 as a potential oncogenic transcript and promising prognostic biomarker in certain cancers, however, its precise functions and underlying molecular mechanisms remains to be elucidated. Thus far, few studies with regard to PVT1 were done in the context of ccRCC, the functions and molecular mechanisms of PVT1 were largely unknown to us. Wu et al. assessed 82 cancer-associated lncRNAs in 71 ccRCC patients by RT-PCR in paired tissues and serum, they identified a serum five-lncRNAs-panel including PVT1 of clinical value in discriminating patients with ccRCC from healthy controls [13]. Since PVT1 can act as an important oncogenic driver in many cancers, and it may also help identify ccRCC patients as serum biomarker, we are eager to figure out whether it can play important roles in the carcinogenesis of ccRCC and why it can exert regulatory functions in ccRCC.

In an attempt to elucidate the roles and downstream events of PVT1 in ccRCC, we firstly evaluated the expression level and clinicopathologic significance of PVT1 via bioinformatic database and PCR validation with tumor samples and cell lines. After overexpression or silencing of PVT1, a series of *in vitro* and *in vivo* experiments were conducted to explore the impacts of PVT1 on proliferation, migration, invasion, and tumorigenicity of ccRCC cells. Further bioinformatic analysis, luciferase reporter experiments, and RNA immunoprecipitation assay were carried out to reveal if PVT1 could act as a competing endogenous RNA (ceRNA) to sponge miRNAs thereby indirectly regulating downstream target genes. Our works may provide new insights into the roles and mechanisms of PVT1 in ccRCC and may identify a promising diagnostic predictor and therapeutic target for the management of this urological cancer.

## RESULTS

### PVT1 is upregulated in ccRCC

**Figure 1:**
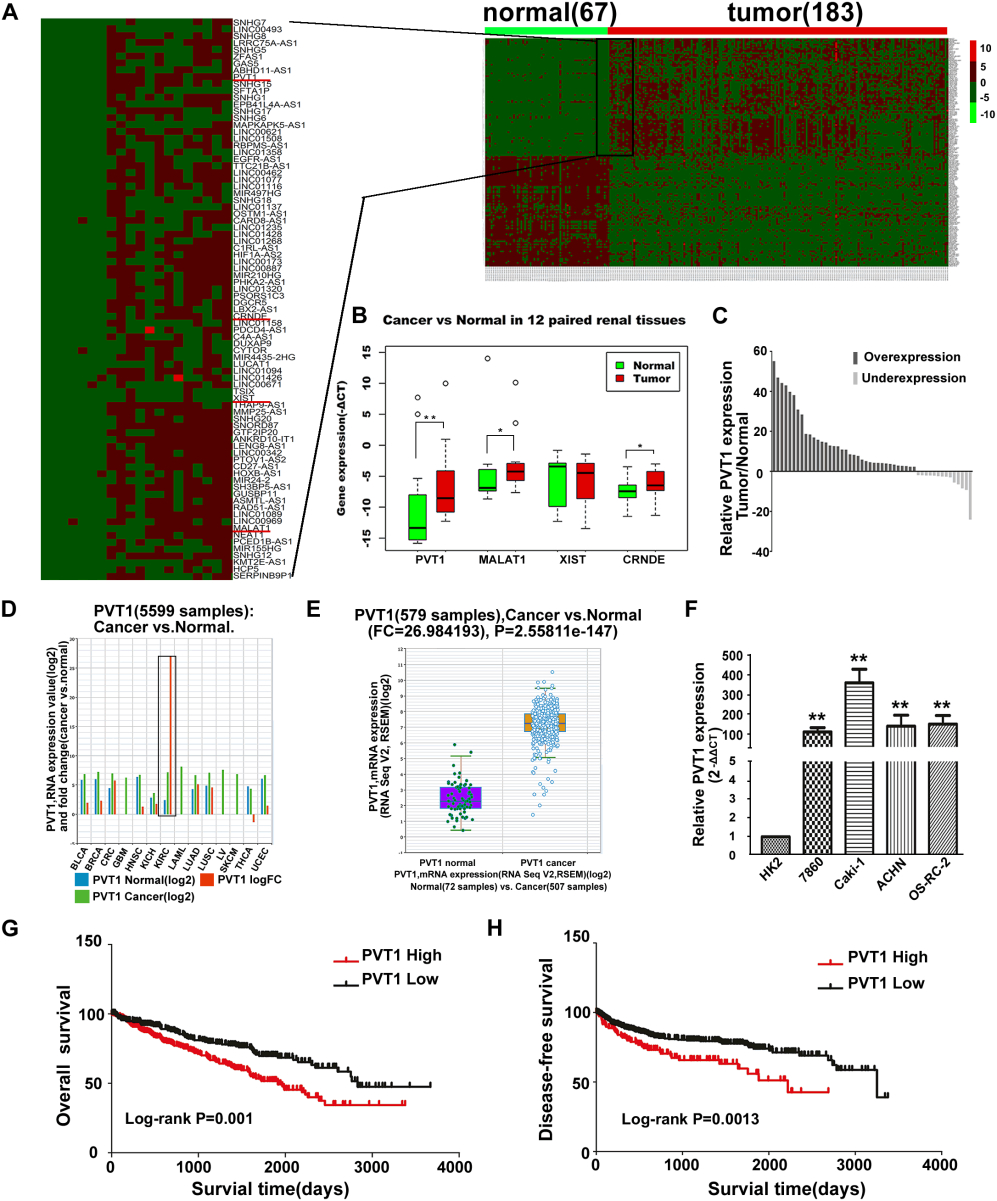
PVT1 was upregulated in ccRCC and correlated with worse overall survival and disease-dree survival in ccRCC patients **(A)** lncRNA expression profile of ccRCC in TCGA database. **(B)** Relative expression of MALAT1, CRNDE, PVT1 and XIST in 12 pairs of ccRCC tumor tissues and their normal counterparts. *, P<0.05, **, P<0.01. **(C)** Relative expression of PVT1 in 50 pairs of ccRCC tumor tissues and their normal counterparts, P<0.001. **(D)** The expression of PVT1 among Pan-Cancer including 14 cancer types from TCGA Data Portal. The black box means the expression of PVT1 in normal or clear cell kidney carcinoma. **(E)** The expression of PVT1 in clear cell renal carcinoma tissues and their normal counterparts from TCGA Data Portal. **(F)** Real-time PCR analysis of PVT1 expression in HK-2 and indicated renal carcinoma cell lines. **(G, H)** Kaplan–Meier overall survival and disease-free survival curves of ccRCC patients according to the expression level of PVT1 in TCGA database (P≤ 0.001, log-rank).

A processed lncRNA expression data in ccRCC from TCGA database was acquired from TANRIC database, and analyzed with the limma package of R. About 159 annotated differential expressed lncRNAs were identified including 82 upregulated and 77 down-expressed ones, among which the expression of some well characterized lncRNA such as MALAT1, CRNDE, PVT1 and XIST were upregulated (Figure 1A). We then examined these four lncRNAs in 12 paired renal cancer tissues and their corresponding noncancerous tissues. This preliminary screening showed MALAT1, CRNDE and PVT1 were significantly upregulated in these 12 paired renal tissues, with PVT1 holding the highest fold change (Figure 1B). Furthermore, we augmented the sample size into 50 patients to validate the expression of PVT1, large-scale sample validation also proved aberrant overexpression of PVT1 in ccRCC (Figure 1C). Besides, the expression of PVT1 in TCGA Data Portal from starBASE v2.0 (http://starbase.sysu.edu.cn/panCancer.php) showed PVT1 is highly expressed in tumor tissues compared with normal tissues in multiple cancers, such as Bladder urothelial carcinoma (BLCA), Breast invasive carcinoma (BRCA), Colon and Rectal adenocarcinoma (CRC), Head and neck squamous cell carcinoma (HNSC), Chromophobe renal cell carcinoma (KICH), Lung adenocarcinoma (LUAD), Glioblastoma multiforme (GBM) and so on. What’s more, the expression of PVT1 in ccRCC is also extremely upregulated (Figure 1D, 1E). We also examined the expression of PVT1 in renal cancer cell lines and immortalized human proximal renal tubule epithelial cell line HK-2 by real-time PCR analysis, which demonstrated that PVT1 expression was consistently upregulated in a panel of 4 human renal cancer cell lines compared to HK-2 (Figure 1F).

### PVT1 expression and clinicopathological factors in ccRCC

PVT1 expression in ccRCC tissues was dichotomized as low or high, with the median level of PVT1 as the cutoff point. While in analyzing the effects of PVT1 expression on DFS, we choose Q3 as the cutoff point to categorized patients into two groups, namely, 25% of the highest expression of PVT1 were arranged as PVT1 High group. Clinicopathological factors were then analyzed in the high and low PVT1 expression groups. As shown in Table 1, in the 50 ccRCC patients of Tongji hospital, highly PVT1 expression was associated with advanced T-stage and Fuhrman grade. As to the TCGA data, highly PVT1 expression was associated with advanced T-stage and tumor–node–metastasis (TNM) stage, histologic grade, and distant metastasis. However, there were no significant correlations between PVT1 expression and other clinicopathological features, such as gender, age or regional lymph node metastasis. As extensive data concerning regional lymph node metastasis remains unclear (273 unsure node status and only 17 cases with positive lymph node metastasis), this may compromise the results of correlation between PVT1 expression and regional lymph node metastasis (Table 2). Kaplan–Meier analysis and log-rank test were used to evaluate the effects of PVT1 expression and clinicopathological factors on OS and DFS in ccRCC patients. The results showed that patients with high expression of PVT1 had reduced OS and DFS compared to those with low PVT1 expression (Figure 1G, 1H). As shown in Table 3, univariate Cox regression analysis indicated that neoplasm histologic grade, TNM stage, distant metastasis, and PVT1 expression were potential predictors for OS and DFS. Besides, multivariate Cox regression analysis indicated that PVT1 was an independent prognostic indicator for OS (hazard ratio (HR)=1.494, 95% confidence interval (CI)=1.081–2.063, p=0.014) in patients with ccRCC. However, its expression was not significant for DFS (HR=1.469, 95% CI=0.976–2.211, p=0.065) in patients with ccRCC. As a result, PVT1 may have the potential to become useful biomarker for diagnosis and survival evaluation of RCC patients.

**Table 1:** Association between PVT1 expression and Clinical characteristics in 50 ccRCC from Tong Ji hospital

Characteristic	Total (n = 50)	PVT1 expression		P
		High	Low	
Gender				
Male	27	15	12	0.19
Female	23	11	12	
Age (years)				
≤60	24	11	13	0.19
>60	26	14	12	
Fuhrman Grade				
I-III	25	8	17	0.00*
III-IV	25	20	5	
TNM stage				
I-II	25	8	17	0.000*
III-IV	25	18	7	

NOTE: Two-tailed fisher’s exact test was done with the SPSS software program to determine the statistical significance of the level of expression of PVT1 in different Clinical characteristics, *P < 0.05 was considered significant.

**Table 2:** Association between PVT1 expression and Clinical characteristics in ccRCC from TCGA Data Portal

Characteristic	Total (n = 528)	PVT1 expression		P
		High	Low	
Gender				
Men	341	178	163	0.203
Women	187	86	101	
Age (years)				
≤60	240	123	117	0.662
>60	288	141	147	
Grade				
G_1_- G_2_	244	110	134	0.027*
G_3_- G_4_	279	153	126	
G_X_	5	1	4	
T-stage				
T1-T2	338	152	186	0.003*
T3-T4	190	112	78	
TNM stage				
I-III	321	140	181	0.000*
III-IV	207	124	83	
Lymph node metastasis				
N_0_	238	114	124	0.381
N_1_	17	11	6	
N_X_	273	138	135	
Distant metastasis				
M_0_	454	199	225	0.017*
M_1_	79	49	30	
M_X_	25	16	9	

NOTE: Two-tailed fisher’s exact test was done with the SPSS software program to determine the statistical significance of the level of expression of PVT1 in different Clinical characteristics, *P < 0.05 was considered significant.

**Table 3:** Univariate and multivariate Cox regression analyses for OS and DFS in patients with ccRCC

Variables	OS	DFS
	HR 95% CI p value	HR 95% CI p value
Univariate analysis		
Age ( >60vs. ≤60 years)	1.787 1.292-2.471 0.000*	1.255 0.865-1.821 0.231
Gender (male vs. female)	1.052 0.786-1.442 0.752	1.382 0.921-2.075 0.114
Neoplasm histologic grade(G3+G4 vs. G1+G2)	2.683 1.889-3.810 0.000*	3.446 2.223-5.343 0.000*
T-stage (T3 + T4 vs. T1 + T2)	3.450 2.525-4.717 0.000*	5.352 3.628-7.896 0.000*
TNM stage (III + IV vs. I + II)	4.287 3.089-5.949 0.000*	7.687 5.025-11.76 0.000*
Distant metastasis (yes vs. no)	4.544 3.303-6.251 0.000*	16.20 10.82-24.27 0.000*
PVT1 expression (high vs. low)	1.811 1.322-2.480 0.000*	1.953 1.318-2.894 0.001*
Multivariate analysis		
Neoplasm histologic grade(G3+G4 vs. G1+G2)	1.652 1.137-2.400 0.008*	1.833 1.158-2.904 0.009*
T-stage (T3 + T4 vs. T1 + T2)	1.003 0.539-1.864 0.994	2.051 1.010-4.166 0.047*
TNM stage (III + IV vs. I + II)	2.314 1.124-4.762 0.023*	1.550 0.657-3.659 0317
Distant metastasis (yes vs. no)	2.273 1.541-3.351 0.000*	9.024 5.505-14.79 0.000*
PVT1 expression (high vs. low)	1.494 1.081-2.063 0.014*	1.469 0.976-2.211 0.065

*, p <0.05.

### PVT1 promotes cell proliferation *in vitro*

**Figure 2:**
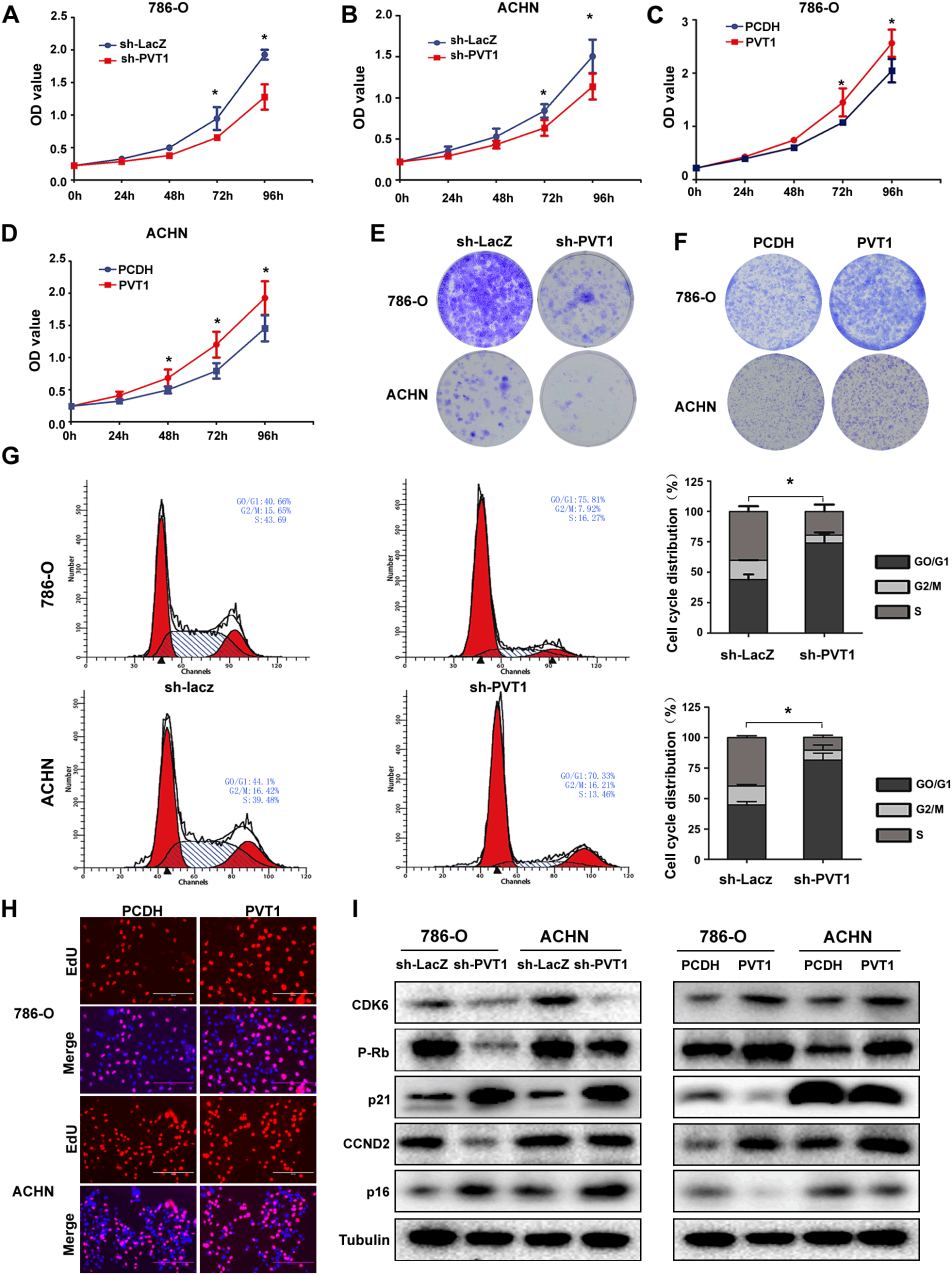
PVT1 promotes cell proliferation *in vitro* **(A, B, C, D)** MTS assays revealed cell growth curves of indicated cells. *, P<0.05. **(E, F)** Representative micrographs of crystal violet-stained cell colonies analyzed by colony formation. **(G)** Flow cytometric analysis showed that PVT1 knockdown increased the number of cells in the G0/G1 phase and reduced the number of cells in the S phase. **(H)** Representative micrographs of EdU incorporation assay in indicated cells. **(I)** The cell cycle related protein levels were tested by western blots.

To explore the role of PVT1 in renal cancer cells, we stably regulated the expression of PVT1 in two RCC cell lines 786-O and ACHN with lenti-viral system, using short hairpin RNA (shRNA) or full-length PVT1 plasmid to silence or overexpress the PVT1 respectively. The nonspecific shRNA (LacZ) or empty plasmid (pCDH) were used as negative control, respectively. The sh-PVT1-1# was used in all the experiments for its knockdown efficiency was better than that of sh-PVT1-2#, while PVT1 overexpression was successful with considerable transfection efficiency in two cell lines (Supplementary Figure 1A, 1B). MTS assay (Figure 2A, 2B, 2C, 2D) and colony formation assay (Figure 2E, 2F) showed that knockdown of PVT1 inhibited cell proliferation in 786-O and ACHN, while PVT1 overexpression promoted cell proliferation. Flow cytometric analysis showed that PVT1 knockdown increased the number of cells in the G0/G1 phase and reduced the number of cells in the S phase (Figure 2G). EdU incorporation assay also validated PVT1 upregulation promoted cancer cell proliferation significantly in statistics (Figure 2H). To further explore the molecular changes which contributed to cell proliferation, we examined several potential proteins involved in the cell cycle progression. As shown in Figure 2I, p-Rb, CDK6 and CCND2 were downregulated, while p21 and p16 was upregulated in PVT1 downregulated cells, while PVT1 overexpression generated opposite changes. All these data demonstrated that PVT1 induced RCC cell proliferation by promoting cell cycle progression, which was in accordance to pro-proliferative functions of PVT1 by the results of GSEA of ccRCC in TCGA database (Supplementary Figure 1C).

### PVT1 promotes cell migration and invasion *in vitro*

**Figure 3:**
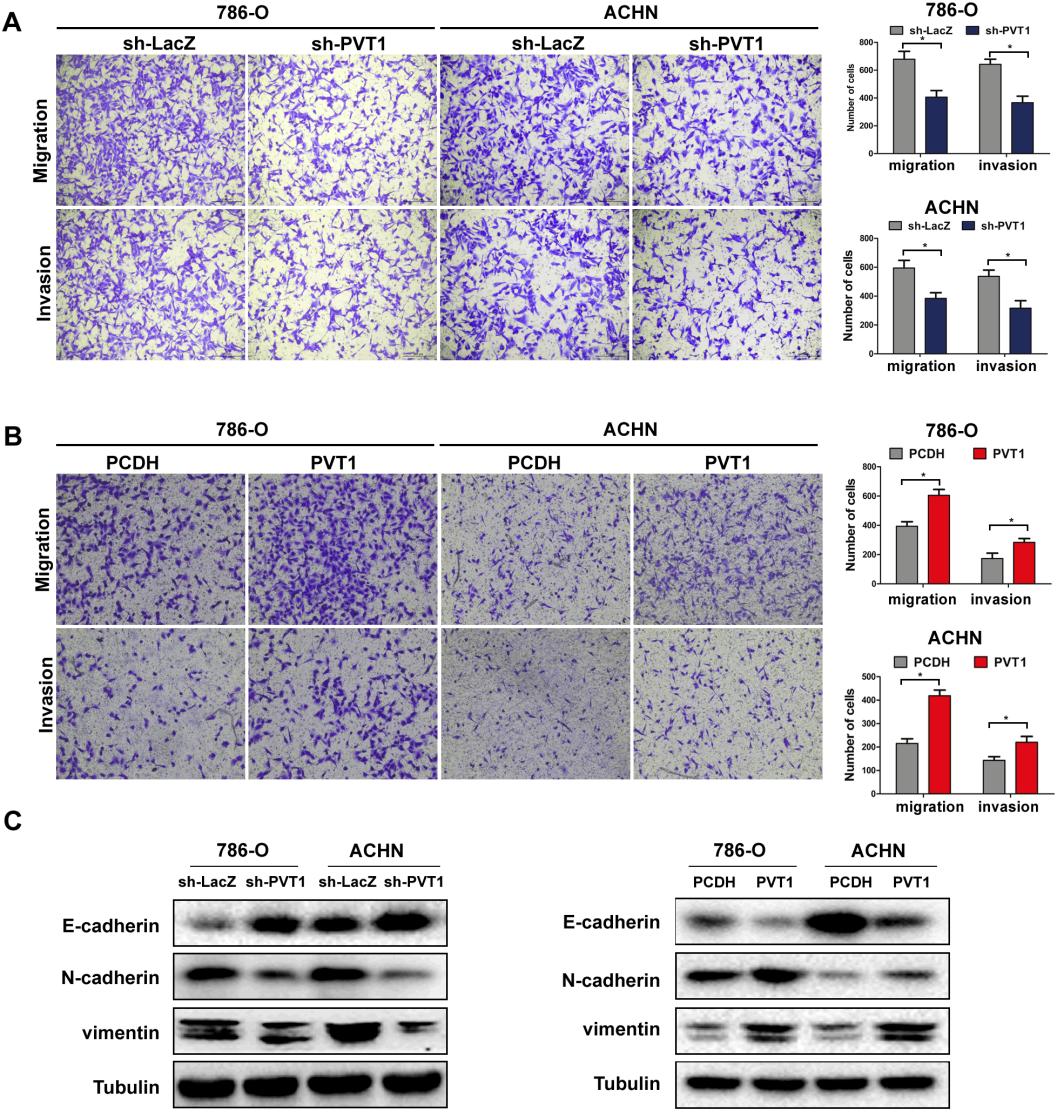
PVT1 promotes cell migration and invasion *in vitro* **(A, B)** Migration and invasion assay for indicated renal cancer cells. Representative photographs were taken at ×200 magnification; number of migrated cells was quantified in ten random images from each treatment group, results were the mean ± SD from three independent experiments and plotted as migrated cell number, *, P<0.05. **(C)** Influence of PVT1 on proteins involved in EMT were tested by western blot.

As epithelial-mesenchymal transition (EMT) is an important enabling property of tumor metastasis, we performed wound healing assay and Transwell assay in 786-O and ACHN cell lines to explore the effect of PVT1 on cell migration and invasion. Wound healing assay (Supplementary Figure 1D, 1E) and Transwell assay showed a higher migratory ability in both cell lines with PVT1 overexpression, while PVT1 knockdown reduced this ability significantly. Similar effects were also observed in Transwell invasion assay (Figure 3A, 3B). Western blot showed that PVT1 silencing lead to the upregulation of epithelial markers E-cadherin but downregulation of mesenchymal markers N-cadherin and vimentin, while ectopic expression of PVT1 turned out to decrease E-cadherin and increase the mesenchymal markers (Figure 3C).

### PVT1 promotes tumor growth and invasion *in vivo*

**Figure 4:**
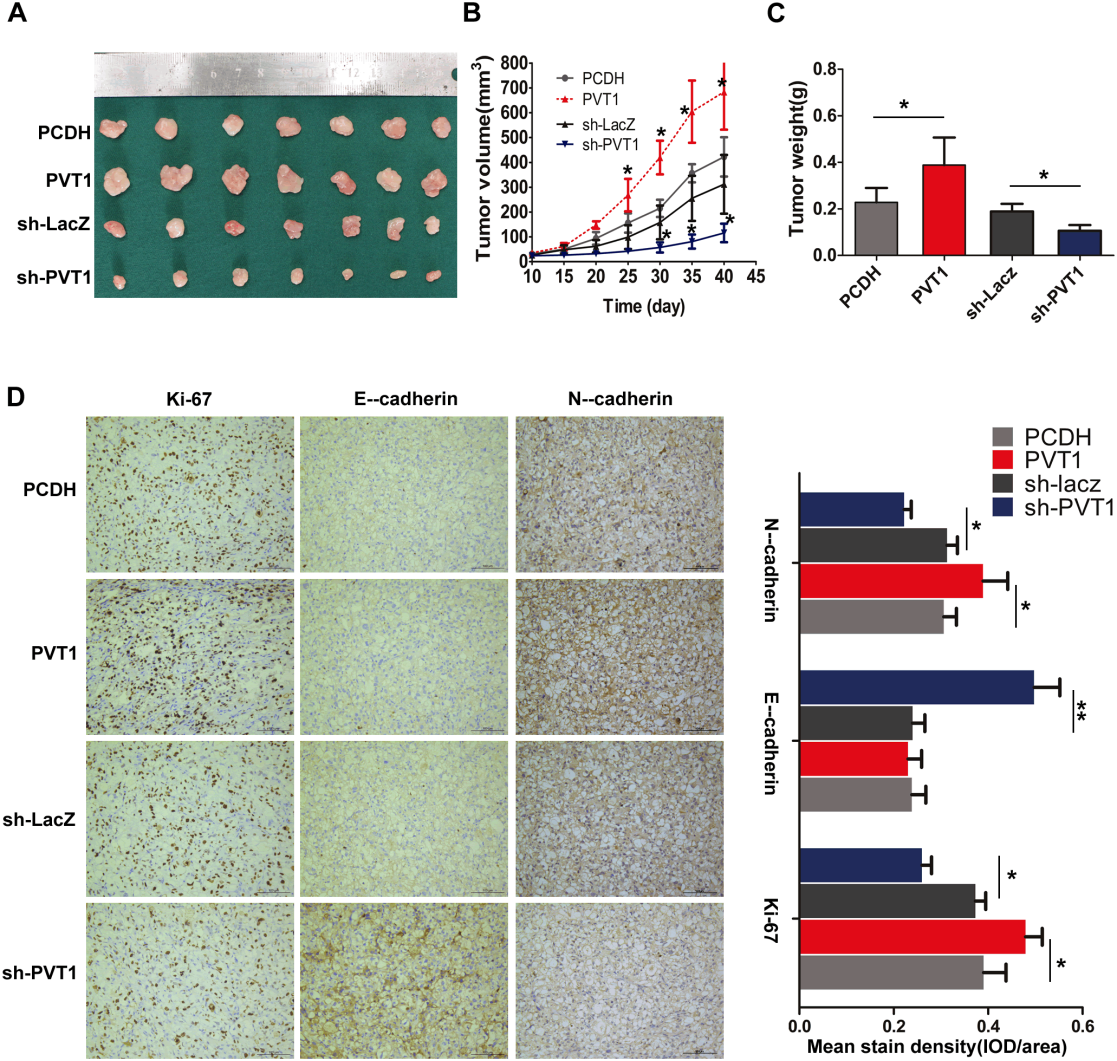
PVT1 promotes cell proliferation and invasion *in vivo* **(A)** Photographs of tumors resected 40 days after inoculation of stably transfected 786-O cells into nude mice. **(B)** Tumor volume were measured by caliper every other 5 days. **(C)** Tumor weight of the four group of nude mouse at the end of 40 days. **(D)** Representative results of the immunohistochemical analysis for Ki-67, E-cadherin and N-cadherin in tumor sections, scale bars represent 100 mm, columns on the right are mean ± SD of mean stain density from five samples of each group. *, P<0.05; **, P<0.01.

To study the effect of PVT1 on the proliferation and invasion of renal cancer cells *in vivo*, 786-O cells transfected with the pCDH or PVT1 overexpression plasmid, sh-LacZ or sh-PVT1 were used in Balb/c nude mice xenograft model. A dramatic increase in tumor volume and weight was observed in the PVT1 group compared with pCDH group, while sh-PVT1 group showed relatively significant decrease in tumor volume and weight (Figure 4A, 4B, 4C). Next, immunostaining analysis of the proliferation marker Ki-67 and EMT-related E-cadherin and N-cadherin was performed in resected tumor tissues. Xenograft tumor derived from PVT1 group showed significantly higher Ki-67 and N-cadherin positive rate compared with that from pCDH control group. Comparatively, sh-PVT1 group showed significantly reduced positivity in contrast to sh-LacZ control group. With regards to E-cadherin, the sh-PVT1 group showed the highest positive rate than other groups. However, we did not observe significant difference of E-cadherin between PVT1 group and control, which may result from low intrinsic E-cadherin expression in 786-O cells (Figure 4D). These results suggest that PVT1 can promote tumor growth and invasion capacity of RCC cells *in vivo*.

### MiR-200s bind to and interact with PVT1

**Figure 5:**
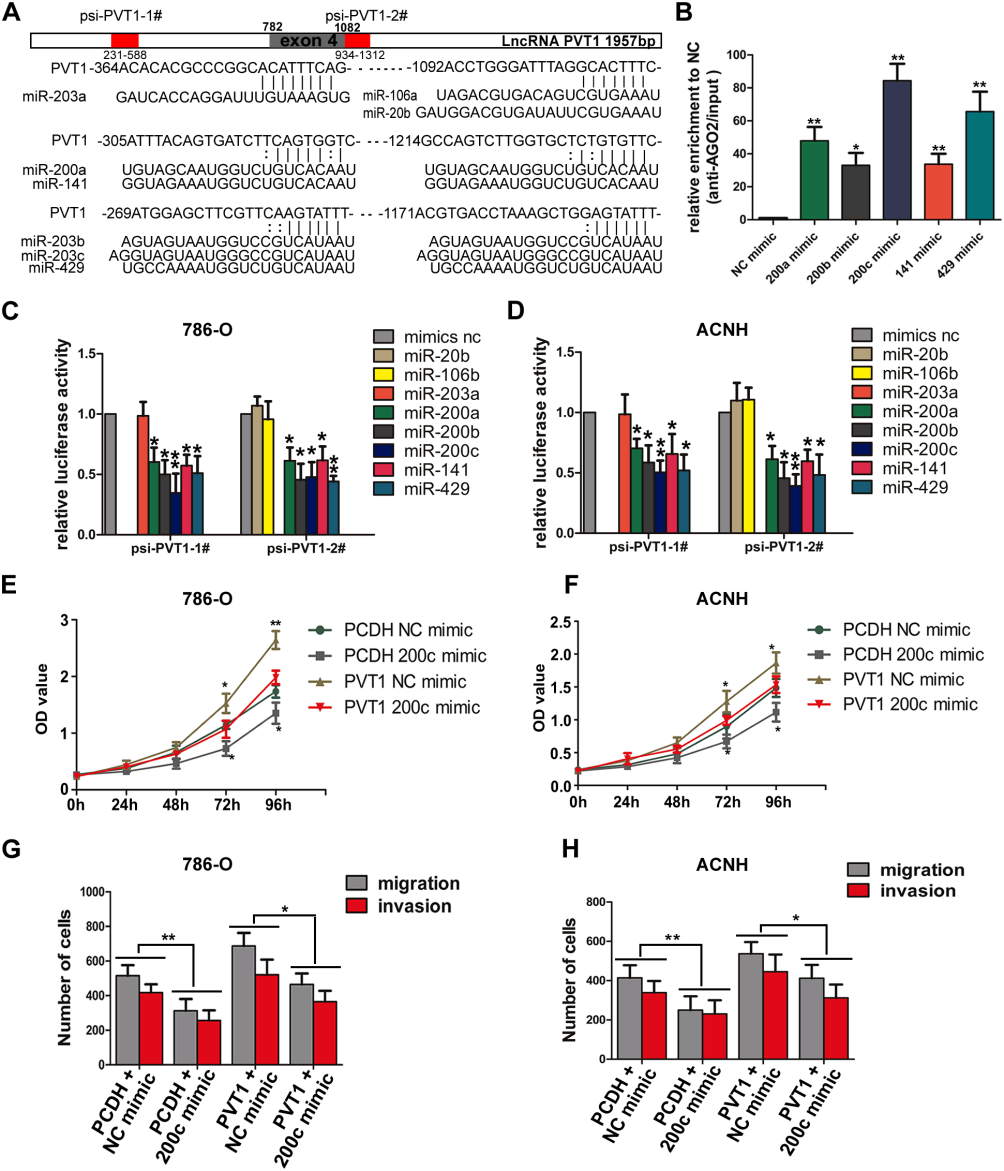
miR-200s bind to PVT1 **(A)** Schematic relative miRNA putative target sites in PVT1. The red part in the column contained the possibly binding sites in PVT1 were named psi-PVT1-1# and psi-PVT1-2#, the gray part stood for the 4th exon of PVT1. **(B)** Anti-AGO2 RIP was performed in 786-O cells transiently overexpressing miR-200s and mimic NC, followed by RT-PCR to detect PVT1 associated with AGO2. (**C** and **D**) Luciferase activity in 786-O and ACHN cells co-transfected with miR-20b/106b-5p/203a/miR-200s/mimic NC and luciferase reporters psi-PVT1-1#/psi-PVT1-2#. Data are presented as the relative ratio of firefly luciferase activity to renilla luciferase activity. Data are shown as mean ± SD from three independent experiments. *, P<0.05, **, P<0.01. **(E, F)** The proliferation assays were performed to evaluate the effect of PVT1 on the function of miR-200c. (**G**, **H**) The migration and invasion assays were performed to evaluate the effect of PVT1 on the function of miR-200c.

The competing endogenous RNA (ceRNA) mechanism indicates that specific lncRNA can serve as sponge for certain active miRNAs, resulting in liberating target mRNA transcripts from miRNAs-associated degradation. Many lncRNAs were found to function as ceRNAs to regulate related target genes through competitively binding microRNAs [14–16]. To determine whether PVT1 can operate as a ceRNA, we analyzed RNA-seq and miRNA-seq data of ccRCC in TCGA database to identify miRNAs with negative correlation with PVT1 expression and binding sites to PVT1 at the same time. Through starBASE 2.0 [17] and RNA22 v2 [18], we found miR-20b/106b-5p/203a and miR-200 family (miR-200a, miR-200b, miR-200c, miR-141, miR-429) were qualified miRNAs that potentially bind to PVT1 (Supplementary Figure 2A). We constructed two luciferase reporters containing those miRNA binding sites in PVT1, namely psi-PVT1-1# and psi-PVT1-2# (Figure 5A). After co-transfecting cells with psi-PVT1-1# or psi-PVT1-2# and miR-20b/106b-5p/203a or miR-200s, we found that overexpression of miR-200s reduced the relative luciferase activities of both reporter vectors, while there was no significant reduction in luciferase activity of miR-20b/106b-5p/miR-203a transfected cells in neither luciferase reporters (Figure 5C, 5D). We then performed anti-AGO2 RNA immunoprecipitation assay by transfecting miR-200s mimics and pulling down endogenous PVT1 via AGO2 in 786-O cells. Results shows that miR-200s mimics transfection increased PVT1 binding with AGO2, with the highest fold change in miR-200c transfected cells (Figure 5B). Besides, PVT1 overexpression downregulated the expression of miR-200s but not that of other miRNAs in both cell lines (Supplementary Figure 2B, 2C). As proper intracellular localization of lncRNA is the prerequisite of ceRNA network, we separated the cytoplasmic RNA and nuclear RNA of 786-O cells and detected the subcellular expression of PVT1, NEAT1, XIST, with GAPDH and U6 as cytoplasmic or nuclear control respectively. RT-PCR results indicated PVT1 mainly expressed in cytosol and NEAT1, XIST mainly expressed in nucleus (Supplementary Figure 3). All these results affirmed that miR-200s could bind to and interact with PVT1.

### PVT1 upregulates BMI1, ZEB1 and ZEB2 levels by sponging miR-200s

**Figure 6:**
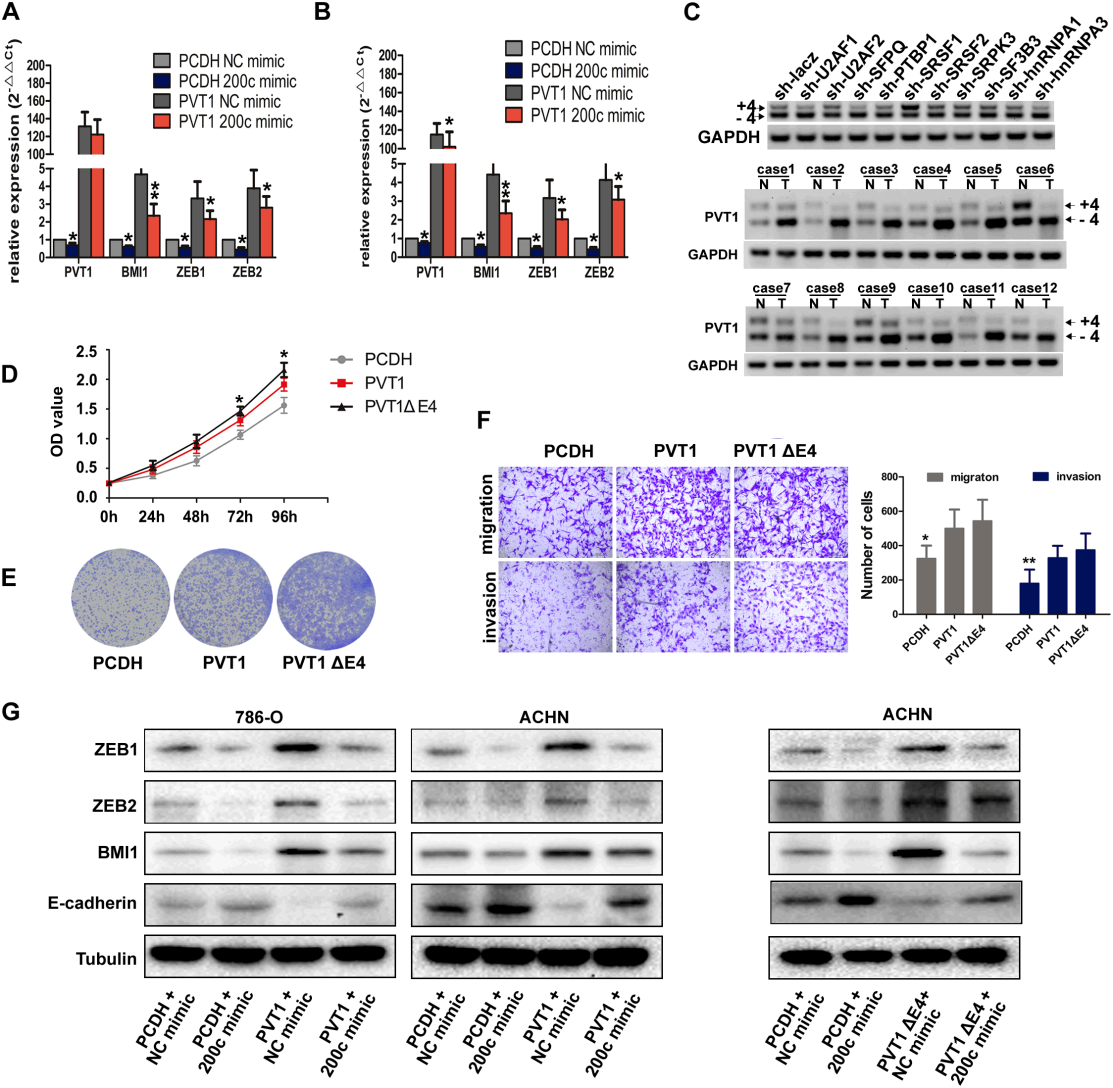
PVT1 and its splicing variant PVT1ΔE4 can both regulate BMI1, ZEB1 and ZEB2 **(A, B, G)** The mRNA (A and B) or protein (G) levels of BMI1, ZEB1 and ZEB2 in indicated 786-O and ACHN cells. **(C)** PCR analysis for ACHN cells stably expressing indicated shRNAs was performed to screen the splicing factors that can regulate PVT1 exon 4 alternative splicing (upper), the Expression of PVT1-4+ and PVT1-4- mRNA in 12 paired renal cancer tissues and adjacent non-tumor tissues by RT-PCR (middle and bottom), GAPDH transcript level was used as the load control. **(D)** MTS assays revealed cell growth curves of 786-O cells stably expressing PVT1ΔE4 transcript and the full length transcript. *, P<0.05, **, P<0.01. **(E)** Representative photographs of crystal violet-stained cell colonies analyzed by colony formation in 786-O cells. **(F)** Representative photographs of migration and invasion assay for indicated 786-O cells, representative photographs were taken at ×200 magnification.

Because PVT1 shares identical sequence with seed sequence of BMI1, ZEB1 and ZEB2 mRNA for complementary pairing with miR-200s, we wondered whether PVT1 could modulate BMI1, ZEB1 and ZEB2 expression. Although PVT1 expression was upregulated in RCC cell lines compared to HK-2 cell, its endogenous expression was relatively low. We transfected PVT1 overexpression plasmid and miR-200c mimic to perform the rescue experiments. Overexpression of PVT1 increased both mRNA and protein level of BMI1, ZEB1 and ZEB2, but ectopic expression of miR-200c abrogated this increase (Figure 6A, 6B, 6G), and also reversed the promotion in proliferation and invasion ability in 786-O and ACHN cells (Figure 5E, 5F, 5G, 5H). We also found a positive correlation between PVT1 and BMI1, ZEB1 and ZEB2 as well as a negative correlation between miR-200c and PVT1, BMI1, ZEB1 and ZEB2 in our 50 paired renal cancer tissues through RT-PCR, which was consistent with that of the analysis from TCGA Data Portal (Supplementary Figure 4A, 4B). Additionally, PVT1 overexpression also increased the expression of BMI1, ZEB1 and ZEB2 in xenograft tumor derived from 786-O cells (Supplementary Figure 4C).

### PVT1ΔE4, a novel splicing variant of PVT1, exerts oncogenic functions through similar ceRNA mechanism

What’s more, we identified a new transcript of PVT1 with its exon 4 skipped during the construction of the overexpression plasmid, which we named it as PVT1ΔE4 subsequently. This transcript exhibited a higher expression level than that of the full-length transcript in ccRCC (Figure 6C) and could also promote cell proliferation and motility in both 786-O and ACHN cells. What’s more, the ability of proliferation in PVT1ΔE4 overexpressed 786-O cell was stronger than that of cell overexpressed with full transcript. However, there was no significant difference in cell mobility between these two transcripts. (Figure 6D, 6E, 6F; Supplementary Figure 5). Through screening a series of splicing factors as we did before in ACHN [19], we found SRSF1 was responsible for exon 4 inclusion/skipping mechanism. SRSF1 interference reduced PVT1ΔE4 and elevated the full-length transcript of PVT1, SRSF1 decreased the inclusion and increased the skipping of exon 4 of full-length transcript and increased the relative expression of PVT1ΔE4 in ccRCC (Figure 6C). Because the 4th exon of PVT1 does not contain the binding site of miR-200s, our results found it could also bind with miR-200s to act as a ceRNA to regulate BMI1, ZEB1 and ZEB2 expression (Figure 6G). All these results suggest a novel PVT1 splicing variant can also regulate BMI1, ZEB1 and ZEB2 by competitively binding miR-200s to exert oncogenic functions, although the splicing mechanisms need further exploration.

## DISCUSSION

Genome-wide sequencing technologies have led to the identification of many noncoding RNAs with important roles in cancer. We found PVT1 was highly upregulated through lncRNA expression profile of ccRCC in TCGA database, and its expression also correlated with worse clinical prognosis. PVT1 was found to function as an oncogene in multiple cancers [9, 20, 21] and overexpression of PVT1 may be a powerful predictor of tumor progression and worse prognosis in a wide range of cancers including non-small cell lung cancer, gastric cancer, bladder cancer, colorectal cancers, pancreatic cancer and so on [10–13, 22–24]. However, its tumorigenic functions and corresponding downstream signaling pathways in ccRCC remains to be elucidated. In this study, we proved that PVT1 is upregulated in both renal cancer tissues and cell lines, and it is correlated with a variety of clinicopathological factors including TNM staging and distant metastasis. We also identified the oncogenic function of PVT1 in renal cancer cells.

The miRNA response elements (MREs) from both lncRNA and target mRNA can bind to certain miRNA, so lncRNAs can act as RNA sponges for specific miRNAs, thus preventing target mRNAs from degradation by these miRNAs. CeRNA was proved to be an effective post-transcriptional regulation mechanism of lncRNAs on downstream target transcripts. By acting as ceRNA, lncRNA-PTENP1 could sponge miR-21 and protect the tumor suppressor PTEN from repressing by miR-21 in ccRCC [25]. Besides, MALAT1 could bind miR-200s to regulate ZEB2 in ccRCC [26], while LncRNA-ATB could regulate ZEB1 and ZEB2 by competitively binding the miR-200s to control EMT and invasion in hepatoma cells [27]. We wondered whether PVT1 might also function as a ceRNA to promote tumorigenesis in ccRCC through some miRNAs, so we analyzed RNA-seq and miRNA-seq data of ccRCC in TCGA database to identify miRNAs negatively correlated with PVT1 expression and bearing binding sites to PVT1 as well. Through starBASE 2.0 and RNA22 v2, we found miR-20b/106b-5p/203a and miR-200s are qualified potential miRNAs that can bind to PVT1.

Paola et al. analyzed miRNA-mediated sponge interactions (MMI-networks) using breast cancer expression data provided by TCGA. They found PVT1 revealed a net binding preference towards the miR-200 family as the bone of contention with its rival mRNAs. But this competing mechanism only works in normal tissues, while it is abolished in tumor context as miR-200s were upregulated in breast tumor tissues, and this could also explain the upregulation of E-cadherin in breast tumor tissues [28]. On the contrary, PVT1 was significantly upregulated while miR-200s were downregulated in ccRCC tumor tissues, and this implies the possibility for PVT1 to bind miR-200s and act as a ceRNA in ccRCC.

We proved PVT1 could bind with miR-200s especially with miR-200c through luciferase report assay and anti-AGO2 RIP, while miR-20b/106b-5p and miR-203a didn’t seem to bind with PVT1 as they didn’t reduce the luciferase intensity of report vectors. With the members of miR-200a, miR-200b, miR-200c, miR-141 and miR-429, miR-200s is one of the best studied miRNA families which mainly involved in EMT process [29–31]. Qiu et al. demonstrated that miR-429 could suppresses cell proliferation, EMT, and metastasis by directly targeting of BMI1 and E2F3 in renal cell carcinoma [32]. Our functional rescue assays indicated that PVT1 overexpression increased both mRNA and protein level of BMI1, ZEB1 and ZEB2, however, ectopic expression of miR-200c can abrogate this increase in both the mesenchymal markers and tumorigenic properties such as proliferation and invasion. Also, in the animal experiments, PVT1 overexpression increased the expression of BMI1, ZEB1 and ZEB2 in xenograft tumor derived from 786-O cells. Further, a positive correlation between PVT1 and BMI1, ZEB1 and ZEB2 was found in our renal samples, which was also consistent with results from TCGA Data Portal. Besides, we also proved that the majority of PVT1 transcript locates in cytoplasm, which permits its execution of duty. These data are consistent with the ceRNA hypothesis that ceRNAs could regulate other genes post-transcriptionally through competitively binding with miRNAs.

Interestingly, we firstly identified and confirmed a new transcript of PVT1 (PVT1ΔE4), and found the endogenous expression of this transcript is even more abundant than its full-length sibling in ccRCC. Furthermore, series of functional experiments also validated its oncogenic roles in renal cancer cells. Despite the deletion defect on the fourth exon, it does not affect its binding with miR-200s and the regulatory capability residing in full-length transcript still remains in PVT1ΔE4. These results implied that the production of this spliced transcript might result from functional redundancy, which further highlighted the pivotal roles of PVT1 in cancers. However, our results also showed that knockdown of oncogenic splicing factor SRSF1 reduced the relative expression of PVT1ΔE4 and increased the full-length one accordingly, so there might also be certain differences between these two transcripts. Further experiments are needed to warrant the elucidation of the specific functions and mechanism of the fourth exon. Our study provided first functional annotation of different lncRNA splicing transcripts in tumor context. Considering that aberrant alternative splicing of pre-mRNA is one of the molecular hallmarks of cancer and the emerging functions of lncRNA [33, 34], the alternative splicing of pre-lncRNA may also have the potential to affect certain cellular processes. Besides, our results showed the spliced transcript was even more abundant in ccRCC, the expression of these two transcripts should be examined in other cancer types. Since most of the studies with regard to PVT1 used the full-length transcript for ectopic expression or RNA pulldown, the results from their studies should be deciphered with additional caution [9, 20].

Taken together, our research demonstrates that PVT1 and its splicing variant PVT1ΔE4 could promote renal cancer cell proliferation and invasion at least partially by competitively binding with miR-200 family through regulating the expression of BMI1, ZEB1 and ZEB2, and overexpression of PVT1 may represent a biomarker of poor prognosis in ccRCC.

## MATERIALS AND METHODS

### Human samples and clinical data from TCGA Data Portal

A total of 50 paired clear cell renal cell carcinoma and corresponding noncancerous tissues were obtained sequentially from patients undergoing radical nephrectomy from the period of 2014–2016 in Tongji hospital, Tongji medical college, Huazhong University of Science and Technology. The patients group consisted of 27 males and 23 females with a median age of 59.8 years (37–75 years). Informed consent was obtained from each patient and the use of tissue samples for all experiments was approved by Ethics Committee of Tongji Hospital. Corresponding noncancerous tissues were acquired at least 5cm away from the tumor site.

Gene expression data and corresponding clinical data of 72 paired normal tissues and 534 tumor tissues in ccRCC patients and relative smaller number of miRNA expression date were downloaded from Cancer Browser of The UCSC Cancer Genomics Browser (https://genome-cancer.ucsc.edu), which supports us a comprehensive, up-to-date and curated TCGA data collection [35]. RNA-seq data of ccRCC patients in TCGA database were also downloaded from TANRIC (The Atlas of Noncoding RNAs in Cancer), a user-friendly, open-access web resource for interactive exploration of lncRNAs in cancer based on recent large-scale RNA-seq datasets, especially those from The Cancer Genome Atlas (TCGA) [36].

### Cell culture, infection, transfection

293T, ACHN and HK-2 cells were maintained in Dulbecco’s modified Eagle’s medium supplemented with 10% fetal bovine serum (FBS) and 2 mmol/L L-glutamine in a humidified atmosphere at 37°C with 5% CO2. 786-O, OS-RC-2 and CaKi-1 cells were cultured in RPMI-1640 supplemented with 10% FBS and 2 mmol/L L-glutamine. Oligos corresponding to the target sequences were annealed and cloned into the HpaI and XhoI sites of the pSicoR plasmid (Addgene). The following target regions were chosen: PVT1-1#, GCUUGGAGGCUGAGGAGUU; PVT1-2#, CCCAACAGGAGGACAGCUU. Two putative PVT1 target sites were cloned into the XhoI-NotI site of the dual luciferase Psi-CHECK2 plasmid (Promega) separately. PVT1 cDNA was PCR-amplified by KOD-Plus-Neo and subcloned into EcoRI and BamHI sites of pCDH lentivector (System Biosciences, USA). All plasmids were verified by sequencing. The primers for vectors construction were provided in Supplementary Table 1, and the details of cell transfection were described in Supplementary Materials.

### Quantitative real-time PCR (qRT-PCR), western blot analysis and immunohistochemistory (IHC)

PVT1 interference efficiency and overexpression efficiency was examined by qRT-PCR using SYBR Premix Ex TaqTM (TaKaRa, Dalian, China). The details of western blot, IHC and antibodies were described in Supplementary Materials. Primers for real-time PCR were listed in the Supplementary Table 2.

### Cell proliferation, migration and invasion assays

Cell viability was assessed at 0, 24, 48, 72 and 96 hours after different treatments by MTS (Sigma, USA) according to the manufacturer’s instructions. The MTS have five replications. The colony formation and EdU assay has three replications. The details of the protocol of EdU assay, colony formation and migratory and invasion assays were included in Supplementary Materials.

### Flow cytometric analysis

Cells transfected with sh-PVT1 or sh-LacZ were seeded in 6-well plates with 2*10^5^ cells per well, the cells were starved for 24 h in serum-free medium before incubated with fresh growth medium for 48h. Then cells were harvested for cycle analysis with Propidium Iodide staining using the BD Cycletest Plus DNA Reagent Kit (BD Biosciences, MA, USA) and analyzed by FACScan flow cytometer (Becton Dickinson, USA). The percentage of the cells in G0–G1, S, and G2–M phases were counted and compared

### Xenograft mouse model using nude mice

The animal experiments were approved by the Animal Care and Use Committee of Tongji Medical College, Huazhong University of Science and Technology. Thirty-two male BALB/c nude mice (4-weeks-old) were randomly divided into four groups, 5×10^6^ 786-O cells were injected subcutaneously into the right flank of each mouse. Tumor volume was calculated every other five days using the formula V = 0.5*ab^2^, where *a* represents the length and *b* represents the width of the tumor. Animals were sacrificed 40 days after inoculation and tumors were weighed.

### Luciferase assays

Briefly, 786-O and ACHN cells were seeded in 48-well plates (5000 cells per well) and co-transfected with 100 ng psiCHECK2 Luciferase vectors containing target genes 3’UTR with 100 nM miRNA mimics or mimic NC. Forty-eight hours after transfection, Dual- Luciferase Reporter Assay (Promega) were performed according to the manufacturer’s instructions.

### Anti-AGO2 RIP

ACHN cells were transfected with miR-200s or miRNA negative control. After 48 hours, cells were used to perform RIP experiments using an AGO2 antibody (Millipore) according to the manufacturer’s instructions [26].

### Statistical analysis

Statistical analyses were performed using SPSS 20.0 software (IBM, SPSS, Chicago, IL, USA) and R3.30. The significance of the differences between groups was calculated by the Student t-test or χ2 test. DFS and OS rates were calculated by the Kaplan–Meier method with the log-rank test applied for comparison. Survival data were evaluated using univariate Cox proportional hazards models.

## SUPPLEMENTARY MATERIALS FIGURES AND TABLES



## Abbreviations

PVT1: Plasmacytoma variant translocation 1
ccRCC: clear cell renal cell carcinoma
RIP: RNA immunoprecipitation
OS: overall survival
DFS: disease free survival
