# Supplementary material for: lncRNA PVT1 and its splicing variant function as competing endogenous RNA to regulate clear cell renal cell carcinoma progression
Source: Oncotarget. 2017 Jul 31;8(49):85353–67. doi: 10.18632/oncotarget.19743 (PMC5689614; doi:10.18632/oncotarget.19743)
Supplement: Supplementary file 1 [file oncotarget-08-85353-s001.pdf]

lncRNA PVT1 and its splicing variant function as competing endogenous RNA to regulate clear cell renal cell carcinoma progression

SUPPLEMENTARY MATERIALS

Plasmids transfection

For stable transfection, 1600ng of target lentiviral transfer vector is cotransfected with 800ng each of the three helper plasmids (Gag-Pol, Rev and VSV-G) into 239T cell with Polyethylenimine MAX 40,000(PolyScience) according to the protocol. After 48h transfection, the Supernatant was filtered with 0.22µm sterile Nihon Millipore filters and added to 786-O and ACHN cells plated in 6 well plates. And the lentivirus Supernatant was exchange with fresh culture medium. Three days later, the transfected cells were subject to 2µg/ml puromycin selection for two weeks and the interference or overexpress efficiency was detected by RT-PCR.

For transient transfection, indicated plasmids or miRNA mimics were transfected into 786-O and ACHN cells with GenMute™ siRNA & DNA Transfection Reagent (signagen) according to manufacturer's instructions

Colony formation and EdU assay

786-O cells and ACHN cells were plated at approximately 1,000 cells per well in 6-well plates for Colony formation and $7*10^4$ in 48 well plates for Edu assay after stable infection with indicated lentivirus. Culture medium was changed every 3 days. Colony formation was analyzed 10 days after infection by staining cells with 0.05% crystal violet solution for 20 min. EdU assay was performed after the cells reached 70%-80% confluence according to the manufacturer's instructions.

Scratch wound healing assay

The cells were inoculated onto six-well plates and cultured at 37°C in a 5% CO2 cell incubator. After the cells reached 70%-80% confluence, cross lines were made using a 200-µL sterile pipette tip. The cells were washed three times with sterile PBS to remove the scratched cells. The cells were continuously cultured in serum-free culture medium. After 0 h and 48 h, the cells were photographed. Cell migration distance = distance at 0 h-distance at 48 h.

Transwell migratory and invasion assays

The 24-well transwell plate with 8 µm pore polycarbonate membrane inserts (Corning, New York, USA) was used to analyze the migration and invasive potential of cells according to manufacturer's protocol with three replications. For invasion assay, the membrane was coated with the matrigel (200 ng/ml) (BD Biosciences, Bedford, MA). 10^5 of ACHN and $3*10^4$ of 786-O cells were seeded, and 12 hours later cells invading into the lower surface of the membrane insert were fixed in 100% methanol, stained with 0.05% crystal violet, and quantified by counting in 10 random fields.

Western blots

For western blot, indicated cells were harvested and Protein was extraction with NP-40 lysate and immunoblot analysis was performed according to standard protocol. Primary antibodies E-cadherin, N-cadherin, Vimentin, P21, P16, CDK6, CCDN2, ZEB1 and ZEB2 (1:1000 dilution; CST), P-Rb and BMI1 (Gentex)and β-Tubulin (1:1000 dilution; Affinity).

Immunohistochemistory (IHC)

Paraffin-embedded, formalin-fixed tissues were immunostained for PCNA proteins. Tissues were deparaffinized, rehydrated, and incubated at room temperature in 0.3% H_2O_2 to block endogenous peroxidase and in blocking solution for nonspecific binding. Primary antibody were applied to sections overnight at 4°C. Afterwards, tissues were incubated with anti-mouse HRP conjugated (Abcam, USA) secondary antibody for 1h at room temperature. Then enzyme development was performed with DAB/H2O2 complex for 10 min at room temperature and in the absence of light which provides a brownish precipitation. The primary Ki-67 antibodies (CST, USA), E-cadherin (CST, USA), N-cadherin (CST, USA), BMI1 (Gentex, USA), ZEB1 (CST, USA) and ZEB2 (CST, USA) was used at working dilution 1:100. Stained (brown) cells were quantified as number of positive cells. To evaluate the intensity of antigen immunoreactivity we examined the percent of

positive staining cells in 10 fields at random per rat under 200×magnification analysied with image-proplus 6.0.

Subcellular RNA isolation and RT-PCR assay

Total cellular RNA of 786-O cells was extracted using Trizol extraction, and the The Cytoplasmic and nuclear RNA of cells was extracted using a PARIS™ kit (Ambion) according to the manufacturer's instructions. For mRNA detection, the cDNA were Synthesis with PrimeScript RT reagent Kit (TaKaRa) and detected with RT-PCR using SYBR Premix Ex TaqTM (TaKaRa). For the detection of U6, cDNA were Synthesis and detected with All-in-One™ miRNA First-Strand cDNA Synthesis Kit (GeneCopoeia)

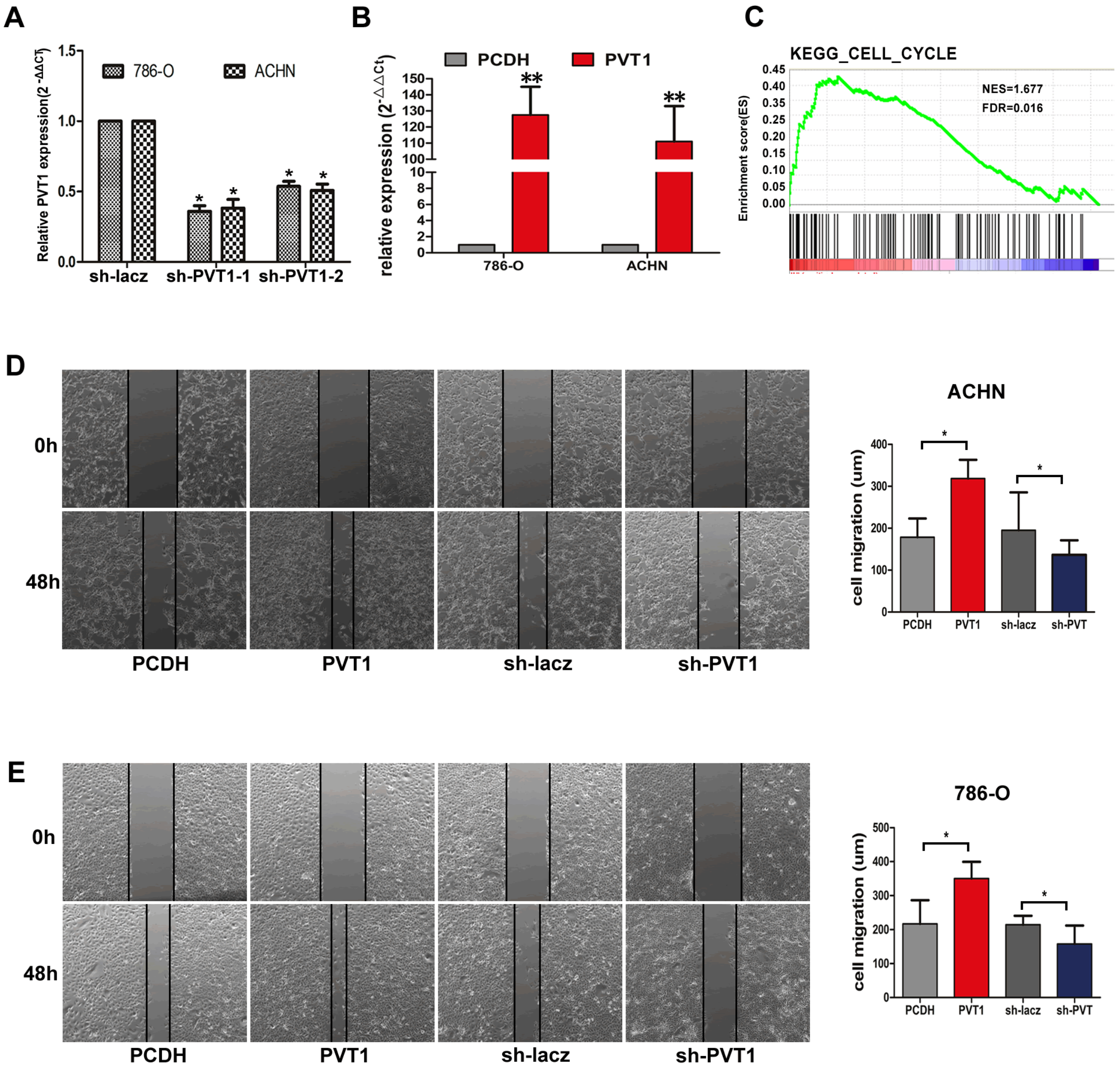

Supplementary Figure 1: PVT1 promotes cell migration *in vitro*. **(A, B)** The efficiency of PVT1 silencing or overexpression in renal cancer cell lines 786-O and ACHN, relative gene expression was determined using the comparative delta-delta CT method ($2^{-\Delta\Delta CT}$). **(C)** GSEA of KEGG_CELL_CYCLE in 50 samples of the highest PVT1 expression versus 50 samples with the lowest PVT1 expression. NES, normalized enrichment score. **(D, E)** Representative micrographs and the statistic migratory distance of Scratch Wound Healing Assay of indicated cells, Data was shown as the means ± SD from three independent experiments*, $p<0.05$.

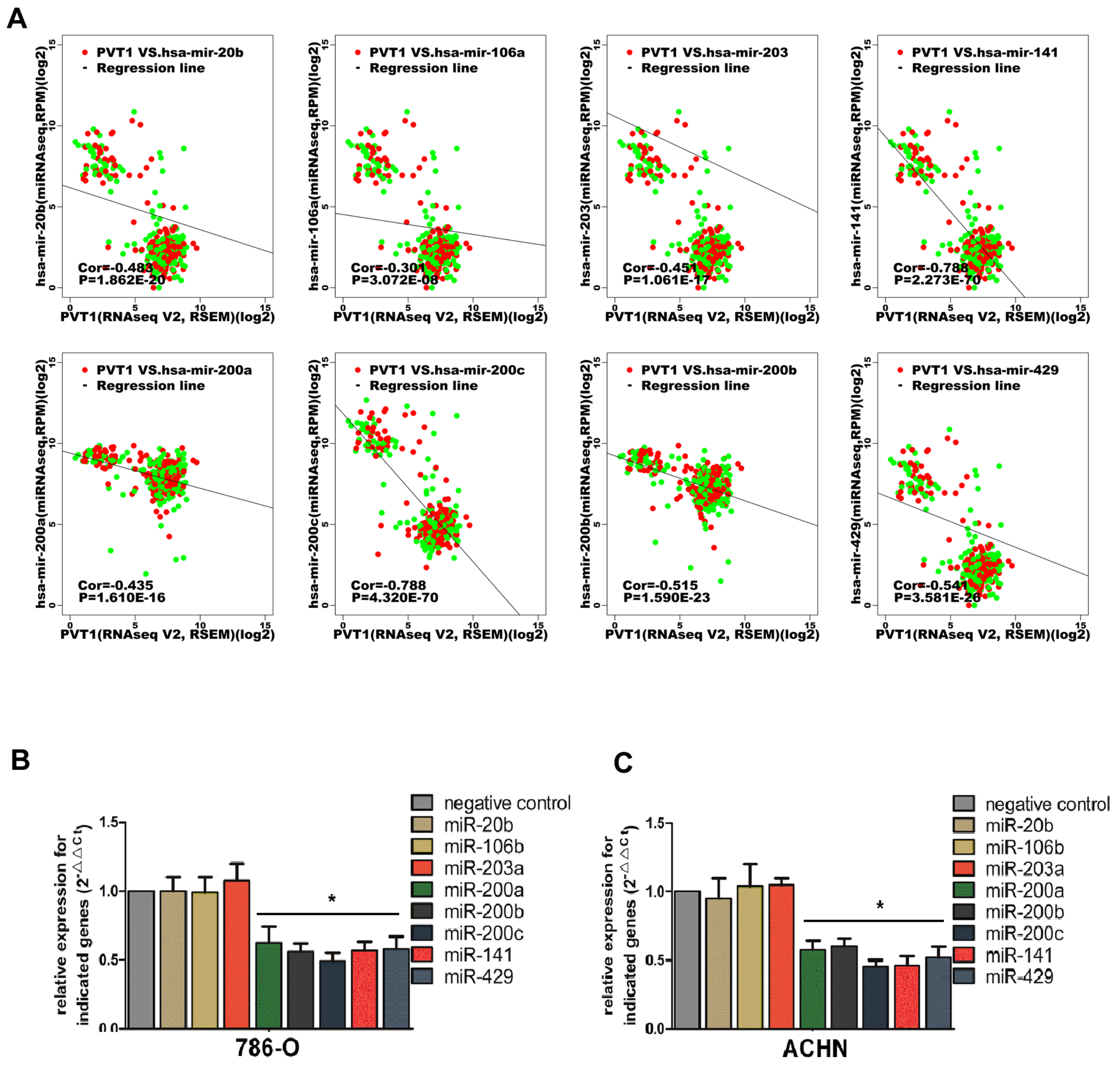

Supplementary Figure 2: Potential interacting miRNAs with PVT1. (A) Pearson correlation analysis of RNA-seq and miRNA-seq of ccRCC in TCGA database to find miRNAs negatively correlated with PVT1 expression and have binding sites of PVT1. **(B, C)** 786-O and ACHN cells were stably transfected with PVT1 over expression plasmid, the expression of indicated miRNAs were detected by RT-PCR.

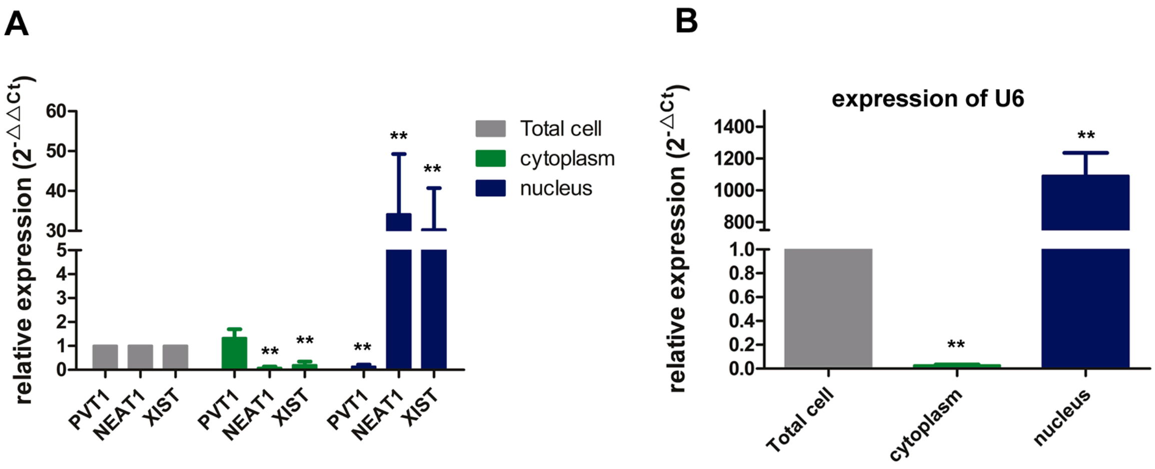

Supplementary Figure 3: Cellular position of PVT1 and its regulation on miR-200s. **(A, B)** The total cell RNA, cytoplasm RNA and nuclear RNA from 786-O cells were separately extracted and synthesized to CDNs respectively. The expression of GAPDH, NEAT1, XIST and PVT1 **(A)** and U6 **(B)** were detected by RT-PCR. Data was shown as the means ± SD from three independent experiments, *, p<0.05, **, p<0.01.

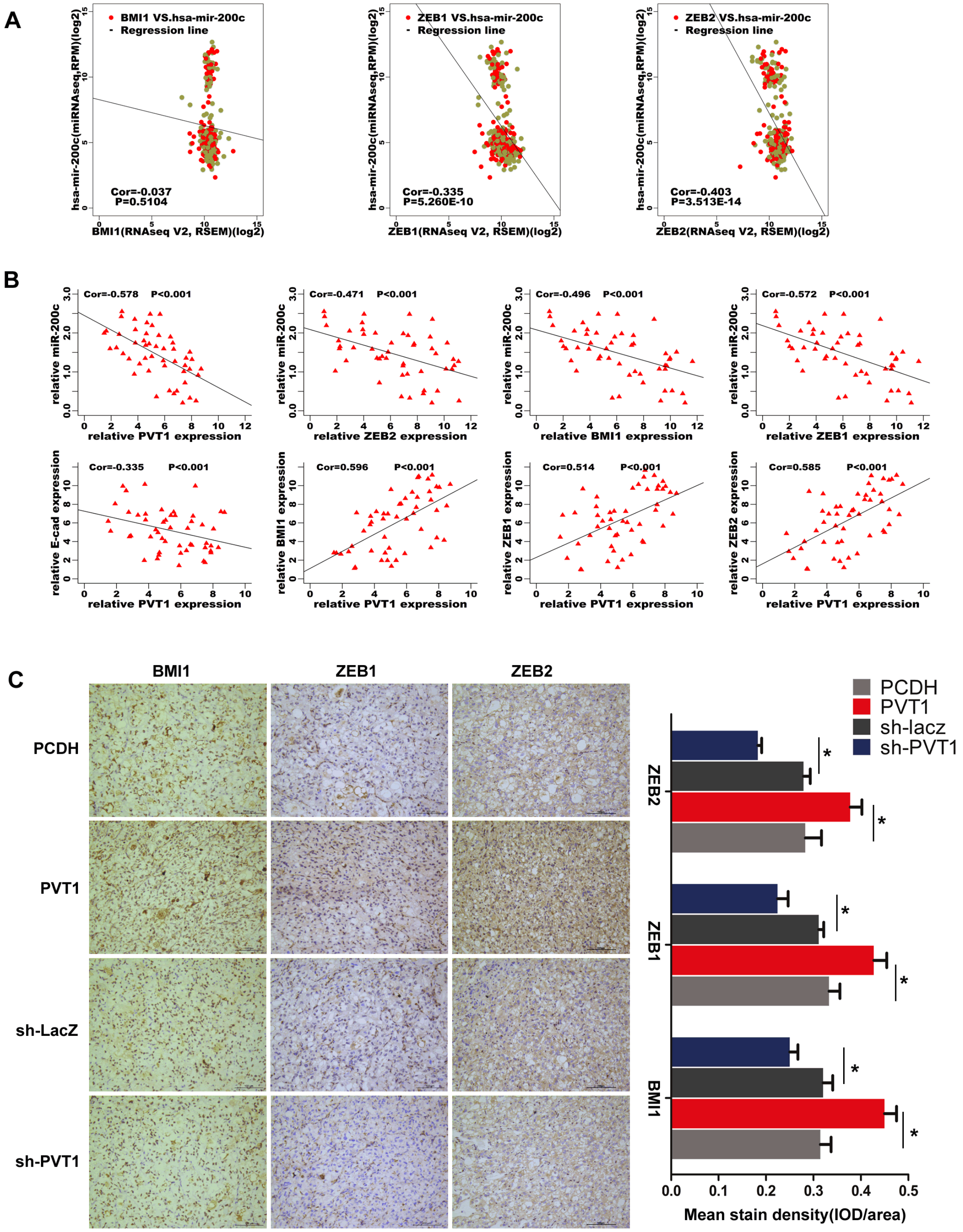

Supplementary Figure 4: PVT1 upregulate BMI1, ZEB1 and ZEB2 levels *in vivo*. (A) Correlation of miR-200c with BMI1, ZEB1 and ZEB2 of ccRCC in TCGA database by Pearson correlation analysis. **(B)** PVT1 expression correlated with BMI1, ZEB1, ZEB2 and ZEB2 in 50 paired clear cell kidney carcinoma tissues and the correlation of miR-200c to BMI1, ZEB1, ZEB2 and PVT1 in 50 paired clear cell kidney carcinoma tissues (by Pearson correlation). **(C)** Representative results of the immunohistochemical analysis for BMI1, ZEB1 and ZEB2 in tumor sections, scale bars represent 100 mm, columns on the right are mean ± SD of mean stain density from five samples of each group, *, p<0.05, **, p<0.01.

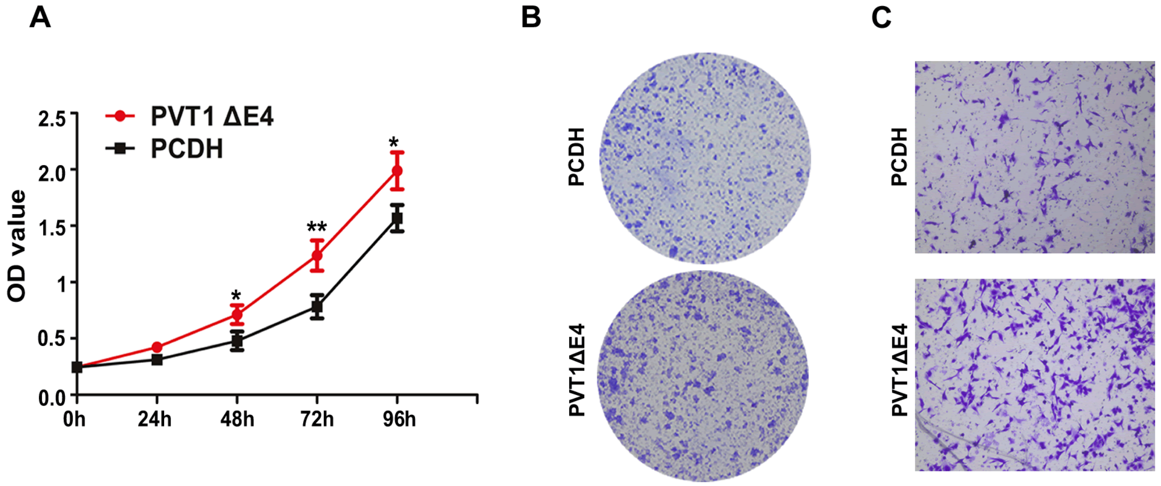

Supplementary Figure 5: PVT1ΔE4 exerts oncogenic functions in renal cancer cells. **(A)** MTS assays revealed cell growth curves of indicated cells, results were the mean ± SD from three independent experiments, *, $P < 0.05$. **(B)** Representative photographs of crystal violet-stained cell colonies analyzed by colony formation in indicated ACHN cells. **(C)** Transwell invasion assay for indicated renal cancer cells. Representative photographs were taken at ×200 magnification; number of migrated cells was quantified in ten random images from each treatment group, results were the mean ± SD from three independent experiments and plotted as migrated cell number, *, $P < 0.05$.

Supplementary Table 1: Primers for vectors construction (underline means restriction site)

Primer name	Primer sequence (5'-3')
psi-PVT1-1#	CCGCTCGAGAAAAGATGCCCCTCAAGATGG
	CGCGCGGCCGCGTCGGGGTCTTACATTCCATA
psi-PVT1-2#	CCGCTCGAGTTTAGTAGAGACGGGGTTTCA
	CGCGCGGCCGCGCAGCAACAGGAGAAGCAA
PVT1	CGGAATTCTCCGGGCAGAGCGCGTGTGG
	CGCGGATCCTAGTAGAAAAAGAATTTATTAGACACGAGG

Supplementary Table 2: Primers for real-time PCR

Primer name	Primer sequence
PVT1	F: CATCCGGCGCTCAGCT
	R: TCATGATGGCTGTATGTGCCA
MALAT1	F: AAAGCAAGGTCTCCCCACAAG
	R: GGTCTGTGCTAGATCAAAAGGCA
CRNDE	F: GTAGAGCCCTTGGAGGTGTTA
	R: TAGCCAGTACAGTAGCTCCAT
XIST	F: CGGGTCTCTTCAAGGACATTTAGCC
	R: GCACCAATACAGAGGAATGGAGGG
BMI1	F:GCTGCCAATGGCTCTAATGAA
ZEB1	R:TGCTGGGCATCGTAAGTATCTT
	F:TTACACCTTTGCATACAGAACCC
	R:TTTACGATTACACCCAGACTGC
ZEB2	F: GCGATGGTCATGCAGTCAG
	R:CAGGTGGCAGGTCATTTTCTT
E-cadherin	F:CGAGAGCTACACGTTCACGG
	R: GGGTGTCGAGGGAAAAATAGG
PVT1(Two transcripts)	F:TGGCTCCCTTGGTGTTCCC
	R:AGCCCGTTATTCTGTCCTTCTCAT
